# Challenges in Estimating the Impact of Vaccination with Sparse Data

**DOI:** 10.1097/EDE.0000000000000938

**Published:** 2018-11-30

**Authors:** Kayoko Shioda, Cynthia Schuck-Paim, Robert J. Taylor, Roger Lustig, Lone Simonsen, Joshua L. Warren, Daniel M. Weinberger

**Affiliations:** From the a Department of Epidemiology of Microbial Diseases, Yale School of Public Health, New Haven, CT; b Sage Analytica, Portland, ME; c Milken Institute School of Public Health, George Washington University, Washington, DC; d Department of Public Health, University of Copenhagen, Denmark; e Department of Biostatistics, Yale School of Public Health, New Haven, CT.

**Keywords:** Bayesian analysis, Brazil, Down-sampling analysis, Hospital admission, Pneumococcal conjugate vaccine, Pneumonia, Sparse data, Synthetic control method, Vaccine program evaluation

## Abstract

**Background:**

The synthetic control model is a powerful tool to quantify the population-level impact of vaccines because it can adjust for trends unrelated to vaccination using a composite of control diseases. Because vaccine impact studies are often conducted using smaller, subnational datasets, we evaluated the performance of synthetic control models with sparse time series data. To obtain more robust estimates of vaccine impacts from noisy time series, we proposed a possible alternative approach, STL+PCA method (seasonal-trend decomposition plus principal component analysis), which first extracts smoothed trends from the control time series and uses them to adjust the outcome.

**Methods:**

Using both the synthetic control and STL+PCA models, we estimated the impact of 10-valent pneumococcal conjugate vaccine on pneumonia hospitalizations among cases <12 months and 80+ years of age during 2004–2014 at the subnational level in Brazil. We compared the performance of these models using simulation analyses.

**Results:**

The synthetic control model was able to adjust for trends unrelated to 10-valent pneumococcal conjugate vaccine in larger states but not in smaller states. Simulation analyses showed that the estimates obtained with the synthetic control approach were biased when there were fewer cases, and only 4% of simulations had credible intervals covering the true estimate. In contrast, the STL+PCA analysis had 90% lower bias and had 95% of simulations, with credible intervals covering the true estimate.

**Conclusions:**

Estimates from the synthetic control model might be biased when data are sparse. The STL+PCA model provides more accurate evaluations of vaccine impact in smaller populations.

## Abstract 2

Supplemental Digital Content is available in the text.

Evaluating vaccination programs is essential to understand their benefit and to guide appropriate allocations of healthcare resources. However, it is challenging to quantify the reduction in disease rates caused by a vaccine at the population level because various unrelated factors also affect the outcomes of interest. For example, improvements in living conditions and access to preventive care may decrease the incidence of a disease and exaggerate the effect of a vaccine. In contrast, improvements in the capacity of inpatient care services and better disease surveillance may increase the number of hospitalizations and thus mask true vaccine-associated declines.^1^

Several methods have been proposed to control for such unrelated trends when assessing the impact of an intervention. Most commonly, linear trends (e.g., interrupted time series analysis,^2^ Holt Winter method^3^) are used to adjust for unrelated changes. A limitation of these approaches is that they assume that the preintervention trends can be described with a limited number of parametric terms and that this trend would continue in the postvaccine period. Alternatively, a time series of a control disease can be used in a regression model to adjust for trends that influence both the disease of interest and the control disease. This approach has the advantage of capturing irregular and unexpected trends, and it draws on information about the control disease from the postintervention period. This approach can be used to isolate the effect of the intervention if the control disease is not influenced by the intervention, if the relationship between the control disease and the disease of interest is consistent over time, and if the control captures the relevant trends. The synthetic control framework builds on this concept but combines several control diseases into a single composite and has been used to assess the impact of interventions in economics, political science, and website analytics.^4–6^ We have previously demonstrated the utility of the synthetic control approach in estimating changes in pneumonia hospitalizations associated with the introduction of pneumococcal conjugate vaccines.^7^

When evaluating the effects of interventions using datasets with large numbers of cases, the time series of the disease of interest and the control diseases are measured with relatively little noise. With these types of data, the synthetic control approach can effectively adjust for unmeasured confounding. In practice, however, interventions often need to be evaluated using noisy data with few cases. It is not clear whether this approach can successfully select an optimal set of controls and adjust for shared underlying trends when using sparser time series data.

We therefore set out to evaluate the performance of the synthetic control approach in such settings.^6,8^ We first evaluated the ability of the method to quantify the impact of a 10-valent pneumococcal conjugate vaccine (PCV10) on all-cause pneumonia hospitalizations at subnational levels in Brazil. Based on these analyses, we found that the synthetic control approach failed to yield reasonable counterfactual estimates when data became sparse. Therefore, we proposed an alternative approach to obtain more robust estimates of vaccine impacts from sparse time series by first extracting smoothed trends from the controls. We evaluated this approach, which we called the STL+PCA method (seasonal-trend decomposition plus principal component analysis), using data from Brazil and using simulated time series data. We compared the performance of STL+PCA with that of the synthetic control approach.

## METHODS

### Hospitalization Data, Down-sampled Data, and Simulated Time Series Data

Three types of data were used in this study: (1) national and state-level hospitalization data in Brazil, (2) down-sampled data based on the national Brazil data, and (3) simulated time series data. Detailed information on these data are provided in eAppendix 1–3; http://links.lww.com/EDE/B425, and the key information for each dataset is highlighted here.

We used a national hospital discharge database from Brazil for hospitalizations that occurred between January 2004 and December 2014.^7^ Hospitalizations were categorized using a single *International Classification of Diseases* (*ICD*) 10 code. There are 27 states in the country, which are grouped into five geographic regions: North (seven states), Northeast (nine states), Southeast (four states), South (three states), and Center-West (four states). Children under 12 months of age and adults 80+ years of age were included in the analyses for contrast because previous studies of national-level data for Brazil demonstrated clear benefits of PCV10 in the infants but no benefit in the elderly.^7,9^ Moreover, there was a strong increasing secular trend in pneumonia hospitalizations among older adults but not among young children (eFigure 1; http://links.lww.com/EDE/B425). These different characteristics provided an opportunity to evaluate the model performance with the presence or absence of the benefits of the vaccine and long-term trends in the time series. The Human Investigation Committee at Yale School of Medicine determined that this research is exempt from review.

To investigate how the performance of various models changes depending on the number of cases per unit time, we performed down-sampling analysis.^10,11^ This approach simulated how the national-level time series would behave had they been drawn from a smaller population. For example, the national population in Brazil is about 200 million, but down-sampling analysis allows us to simulate a time series of a theoretical population of 20 million (i.e., down-sampling rate 10%) or 2 million (i.e., down-sampling rate 1%). Methods are described in detail in eAppendix 2; http://links.lww.com/EDE/B425. In short, we randomly subsampled the time series of *ICD10* chapters from the national-level data, using the binomial distribution with the rates of 10%, 1%, and 0.25%, to simulate the population sizes of different regions and states in Brazil. This sampling process was repeated 100 times for each rate.

We also used simulated monthly time series data, which included an outcome and four control diseases, to demonstrate the performance of various models (eFigure 2; http://links.lww.com/EDE/B425 and eAppendix 3; http://links.lww.com/EDE/B425). The length of time series was 120 months, and a “vaccine-associated” decline was introduced starting in month 85. There was a gradual 20% decline in the number of cases in the outcome that occurred between month 85 and the end of the time series. Both the outcome and control diseases were given an annual seasonality with different amplitude and peak timing, and the time series had a u-shaped curve that (by design) could not be captured by a standard linear trend adjustment. One of the four control diseases was a “perfect” control (blue line in eFigure 2; http://links.lww.com/EDE/B425), which means that the underlying model that generates the mean for the control is the same as the outcome, except the simulated vaccine impact is set to 0. To evaluate how the performance of the statistical models changes depending on the number of cases per unit time, we manipulated the average number of cases for the outcome at the first time point (i.e., intercept) to be roughly 8000 (100%), 800 (10%), 80 (1%), and 20 (0.25%). For each of these sample sizes, we simulated 100 time series data.

### Synthetic Control Model

The synthetic control method has been described previously.^7,12^ The model uses a time series of pneumonia hospitalizations (*ICD10* code: J12–J18) as the outcome and time series of the different control diseases as the covariates; the method relies on Bayesian variable selection to select a set of covariates that jointly explains the outcome best based on the prevaccine data (eAppendix 4; http://links.lww.com/EDE/B425).^7,12^ The covariates (control diseases) were disease categories based on groupings of *ICD10* chapters, such as disease of the circulatory system (I00–I99), skin (L00–L99), musculoskeletal system (M00–M99), and genitourinary system (N00–N99).^7^ The full list of control diseases included in the synthetic control model can be found in eTable 1; http://links.lww.com/EDE/B425. Key assumptions that these control diseases need to satisfy to be valid are (1) they were not affected by PCV10 and (2) relationships between the outcome and control diseases would not change over time had PCV10 not been introduced. We therefore excluded respiratory diseases and other disease categories that could potentially be influenced by PCV10, except for bronchitis/bronchiolitis (J20–J22).^7^

We fit the synthetic control model to monthly data from the prevaccine period and used it to generate a counterfactual prediction for the postvaccine period. Further information can be found in eAppendix 4; http://links.lww.com/EDE/B425, while full details on the synthetic control method are provided in Brodersen et al.^12^ We used the bsts and BoomSpikeSlab packages in R version 3.4.3 (Vienne, Austria) for model fitting.^13,14^ An R script is available on the github repository at https://github.com/weinbergerlab/Brazil_state.

### Seasonal-trend Decomposition Plus Principal Components Analysis Model

To address issues of sparsity in the control variables, we propose an alternative approach, the seasonal-trend decomposition plus principal components analysis model (STL + PCA), where we first extract a long-term trend for each control variable, obtain a “composite” of these trends, and use this composite trend as an explanatory variable in the regression model to generate the counterfactual (eFigure 3; http://links.lww.com/EDE/B425). Therefore, this model does not involve variable selection.

The first step of the STL+PCA method is to extract smoothed trends from the time series of each of the control diseases using the seasonal-trend decomposition procedure based on locally weighted scatterplot smoothing (STL).^15^ The same set of control diseases was used as for the synthetic control model (eTable 1; http://links.lww.com/EDE/B425). The span of the locally weighted scatterplot window can be adjusted to control the smoothness of extracted trends, and we selected the optimal span using the deviance information criterion (eFigure 4; http://links.lww.com/EDE/B425 and eAppendix 5; http://links.lww.com/EDE/B425).^16^

The second step is to obtain a “composite” trend among extracted trends for control diseases and to reduce the dimensionality of the total set of trends for control time series. To do so, we performed a PCA with extracted trends.^17–20^ The first principal component “PC1” is a linear combination of extracted trends for control diseases with maximum variance, which accounted for about 75% to 90% of the total variance in the extracted trends for all control diseases.

In the third and final step, we use PC1 as a covariate in a regression model for the prevaccine data and generate the counterfactual for the postvaccine period. We only included PC1 in the model because PC1 explained most of the variance (75%–90%), and the rest of the principal components mostly captured the remaining noise. More information can be found in eAppendix 5; http://links.lww.com/EDE/B425. We validated the STL+PCA model by performing a cross validation analysis with the prevaccine data (eAppendix 6; http://links.lww.com/EDE/B425). An R script is available on the github repository at https://github.com/weinbergerlab/Brazil_state.

### Evaluation of the Impact of Vaccine

We fit both the synthetic control and STL+PCA models to pneumonia time series from the prevaccine data to establish a relationship between pneumonia and the covariates. We then estimated the number of pneumonia hospitalizations that would have occurred without vaccination in the postvaccine era (i.e., counterfactual), assuming that the relationships between the outcome and covariates were consistent. We calculated the rate ratio (RR) of the observed to the counterfactual pneumonia hospitalizations during the evaluation period. RRs less than one suggest that the vaccine has prevented pneumonia hospitalizations. The evaluation period was 2013–2014 for the Brazil data (i.e., 37–60 months following the introduction of PCV10) and the last two years for the simulated time series data (i.e., 13–36 months after the simulated introduction of the vaccine). Posterior medians and 2.5th and 97.5th percentiles were reported as point estimates and 95% credible intervals (CIs) of RR, respectively.

## RESULTS

### Performance of the Models with National and State-level Data from Brazil

**Figure 1.**
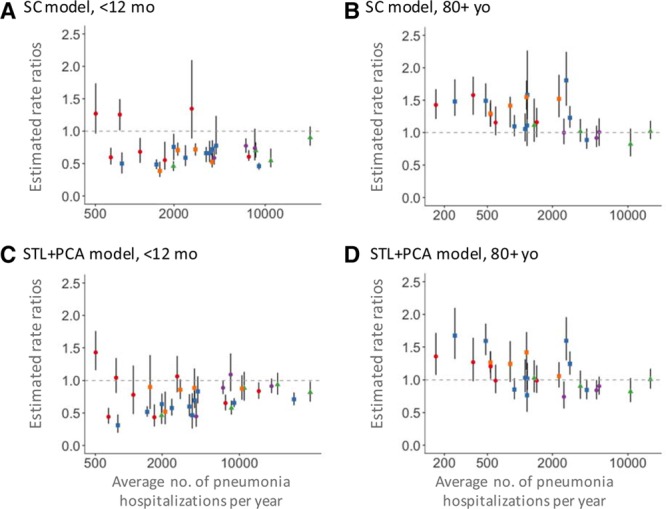
Rate ratios estimated by the synthetic control model are shown in panel A (<12 months of age) and B (80+ years of age). Rate ratios estimated by the STL+PCA model are shown in panel C (<12 months of age) and D (80+ years of age). Estimates of state-level rate ratios by the average number of all-cause pneumonia hospitalizations in Brazil. States in the North region are represented in red, the Northeast region in blue, the Southeast region in green, the South region in purple, and the Center-West region in orange. Rate ratios are the cumulative number of observed pneumonia hospitalizations divided by the cumulative number of counterfactual pneumonia hospitalizations during the evaluation period. PCA indicates principal component analysis; SC, synthetic control; STL, seasonal-trend decomposition procedure based on locally weighted scatterplot smoothing.

The time series for all-cause pneumonia hospitalizations among children under 12 months of age in Brazil showed strong seasonality, with peaks occurring in the winter (eFigure 1A; http://links.lww.com/EDE/B425). Both the synthetic control and STL+PCA models detected a decline in the number of pneumonia hospitalizations after the introduction of PCV10 in 2010 at the national level in this age group. National-level estimates of RR were 0.72 (95% CI = 0.67, 0.77) by the synthetic control model and 0.83 (95% CI = 0.71, 0.96) by the STL+PCA model. The most common control diseases selected in the synthetic control model were bronchitis/bronchiolitis (J20–J22) and malnutrition (E40–E46) for children. The full list of posterior inclusion probabilities of control diseases for children can be found in eAppendix 7; http://links.lww.com/EDE/B426. Similarly, both models estimated declines in most of the states in this age group (Figure 1A and 1C).

Time series data for the older age group showed a complex pattern; all-cause pneumonia hospitalizations, as well as some control diseases, began to increase several years prior to the introduction of PCV10 and continued to increase until the end of the study period (eFigure 1B; http://links.lww.com/EDE/B425). At the national level, both models adjusted for this unexplained long-term increasing trend and found no changes in pneumonia hospitalizations after the introduction of PCV10. Estimated national-level RRs were 0.95 (95% CI = 0.82, 1.12) by the synthetic control model and 1.02 (95% CI = 0.88, 1.18) by the STL+PCA model. The most common control diseases selected in the synthetic control model were other septicemia (A41) and diseases of the circulatory system (I00–I99) for the elderly (eAppendix 8; http://links.lww.com/EDE/B427). When repeating the synthetic control analysis of the elderly at the state level, 12 states (46%) had RR estimates greater than one, which we do not believe is credible in this context. Most of these states were found to have relatively few pneumonia hospitalizations (Figure 1B). When using the STL+PCA approach, fewer states (eight states; 32%) had RRs greater than one. Additionally, the mean squared error (MSE, eAppendix 9; http://links.lww.com/EDE/B425) of state-level RRs compared to the national estimates declined from 0.148 when using the synthetic control model to 0.076 when using STL+PCA (Figure 1D).

### Performance of the Models with Down-sampled Brazil Data

**Figure 2.**
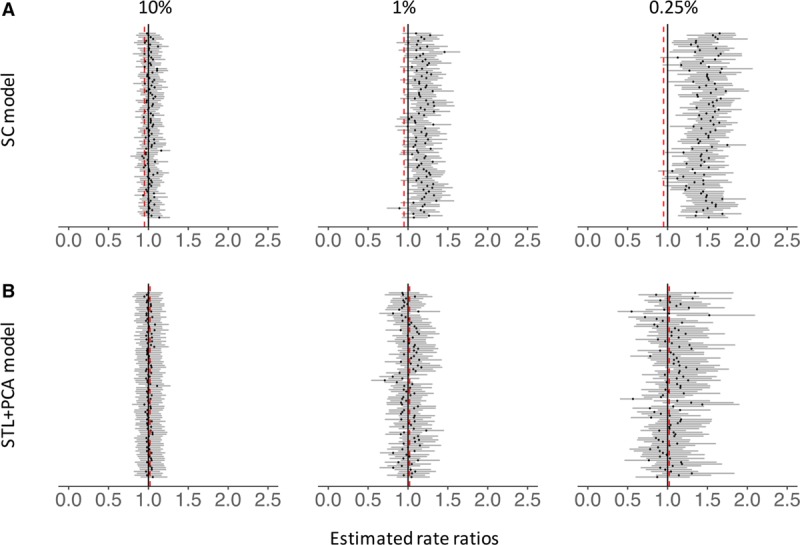
Rate ratios estimated by the synthetic control model (panel A) and the STL+PCA model (panel B). Estimated rate ratios for down-sampled datasets (80+ yo, Brazil). Each black dot represents a RR estimated for each down-sampled dataset. Dark gray bars associated with these dots represent 95% credible intervals for RRs. The percentages at the top represent the down-sampling rates. Black vertical lines represent the null value (RR = 1), and red dashed lines represent national estimates of RR generated by each type of the model. RRs are the cumulative number of observed pneumonia hospitalizations divided by the cumulative number of counterfactual pneumonia hospitalizations during the evaluation period. PCA indicates principal component analysis; RR, rate ratio; SC, synthetic control; STL, seasonal-trend decomposition procedure based on locally weighted scatterplot smoothing.

**Figure 3.**
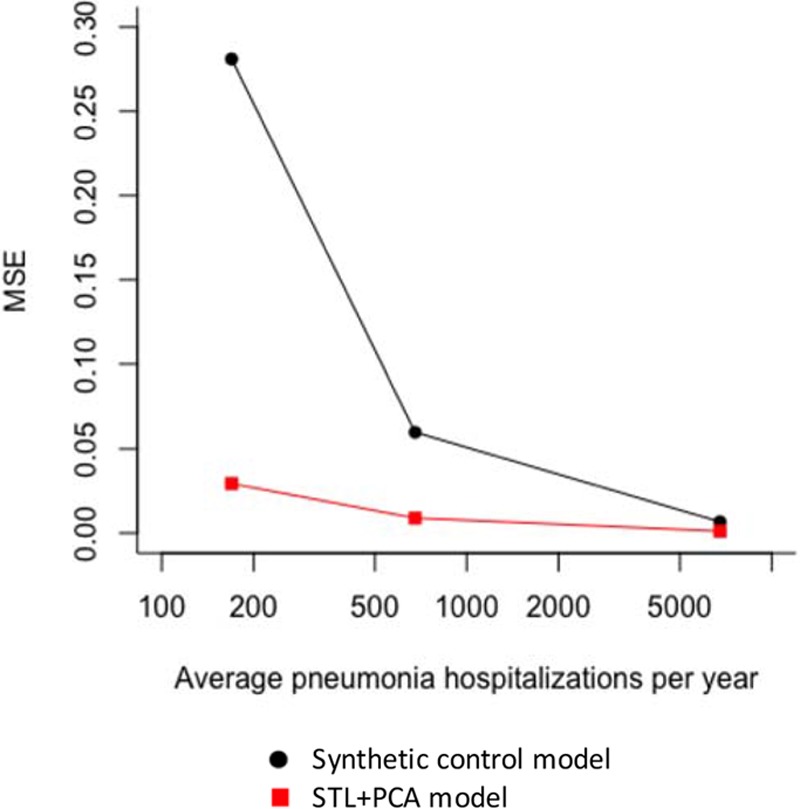
Mean squared errors of estimated rate ratios from down-sampled datasets (80+ yo). MSE, mean squared error; PCA, principal component analysis; STL, seasonal-trend decomposition procedure based on locally weighted scatterplot smoothing.

To further examine the effect of smaller sample size on the accuracy of the estimates from these models while setting aside complicated factors affecting real-world data, we conducted a down-sampling analysis of the national time series from Brazil. For adults of age 80+ years, estimated RRs generated by the synthetic control model rapidly diverged from the national estimate (the “ground truth”), as we sampled successively fewer cases (Figure 2A). When the down-sampling rate was 0.25%, only 4% of the down-sampled datasets covered the national-level RR in their 95% CIs. As a result, MSE increased exponentially as the data became sparse (Figure 3), which was largely driven by increased bias (eFigure 5; http://links.lww.com/EDE/B425). With the national data for the elderly, the synthetic control model selected three control diseases on average (range: one to nine control diseases). However, when the down-sampling rate was 0.25%, the synthetic control model did not select any control diseases in the final model in 47 of the 100 down-sampled datasets (i.e., model only had an intercept and seasonal terms), only one control disease in 49 datasets, and two control diseases in the remaining four datasets. These results demonstrate that the synthetic control model failed to identify an appropriate set of control diseases and thus failed to adjust for unmeasured confounding in the datasets down-sampled to resemble the smaller states. In contrast, the STL+PCA method successfully adjusted for a long-term increasing trend and corrected the bias even when the data became sparse (Figure 2B). Even when the down-sampling rate was 0.25%, 95% of the estimated RRs covered the national-level RR in their 95% CIs. MSEs remained small across all down-sampling rates (Figure 3). When the down-sampling rate was 0.25%, the STL+PCA model had 90% lower MSE than the synthetic control model.

For children less than 12 months of age, where there was no strong secular trend, neither model’s performance was dramatically affected by sample size (eFigure 6; http://links.lww.com/EDE/B425). MSEs remained small, regardless of the down-sampling rate (eFigure 7; http://links.lww.com/EDE/B425). However, the average number of control diseases selected in the synthetic control model was zero in 88 out of 100 down-sampled datasets when the down-sampling rate was 0.25%.

### Performance of the Models with Simulated Time Series Data

**Figure 4.**
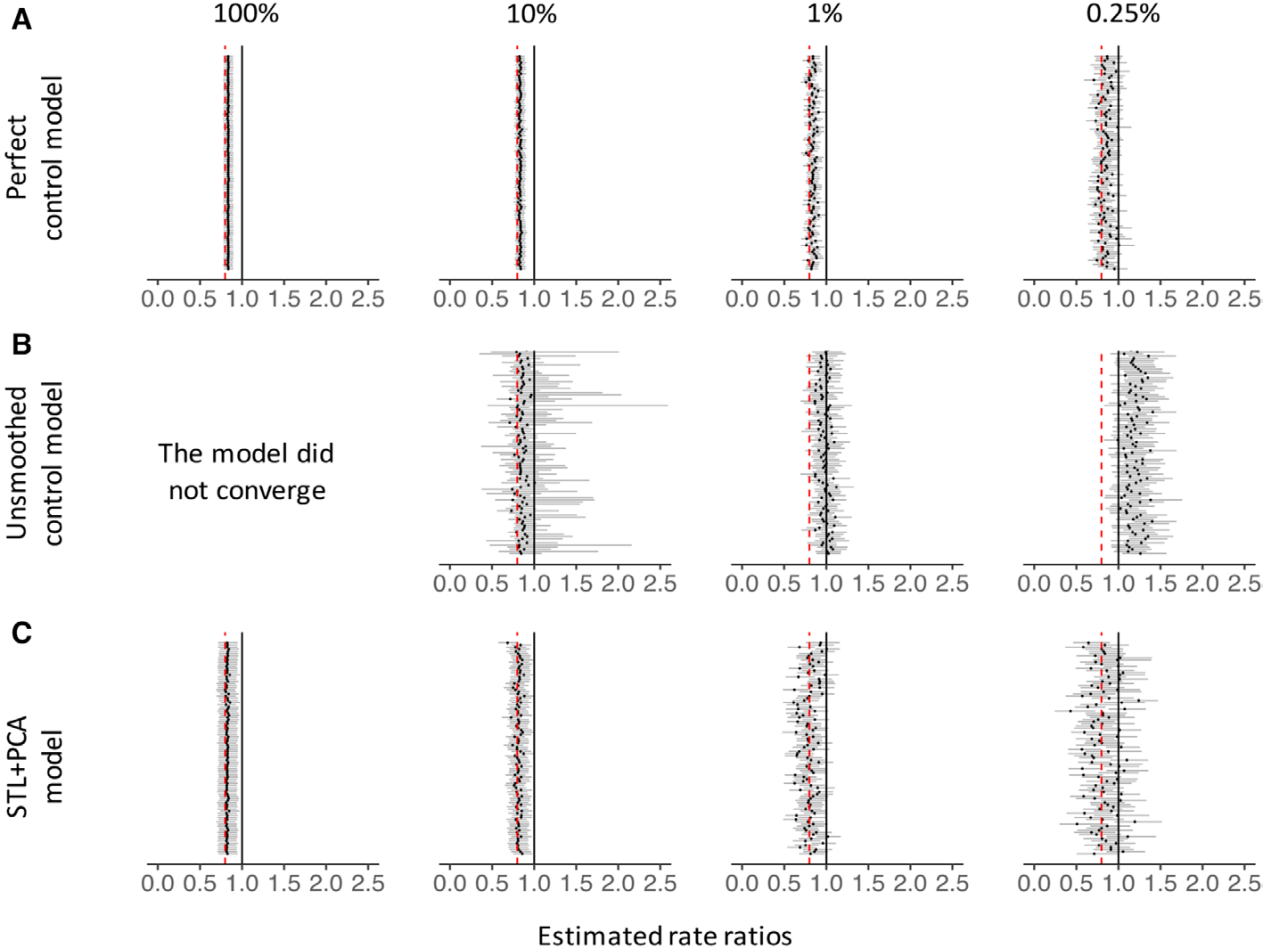
Rate ratios estimated by the perfect control model (panel A), unsmoothed control model (B), and STL+PCA model (C). Estimated rate ratios for simulated time series data. Each black dot represents an RR estimated for each simulated dataset. Dark gray bars associated with these dots represent 95% credible intervals for RRs. The percentages at the top represent the sample size. Black vertical lines represent the null value (RR = 1), and red dashed lines represent the true value of RR (0.8). RRs are the cumulative number of observed pneumonia hospitalizations divided by the cumulative number of counterfactual pneumonia hospitalizations during the evaluation period. PCA indicates principal component analysis; RR, rate ratio; STL, seasonal-trend decomposition procedure based on locally weighted scatterplot smoothing.

Using simulated data, we first fit a model with a single predictor, the “perfect” control, which had the exact same trend as the outcome during the prevaccine period, and tested whether this model was able to recover the true impact of the vaccine (RR = 0.8). Because of this ideal, but not realistic, relationship between the outcome and the predictor, this model was expected to generate the best possible counterfactual. RRs yielded by this model tightly lined up around the true value, even when the data became sparse (Figure 4A). Next, we fit the model which included three controls, but not the perfect control, as predictors (“Unsmoothed control model” in Figure 4B and eFigure 8; http://links.lww.com/EDE/B425). This model failed to converge when the data size was large because of a strong collinearity among those controls. As the data became sparse, estimated RRs moved away from the null and became significantly greater than the true RR (Figure 4B).

The STL+PCA model successfully recovered the true RR, even when the number of cases became smaller. Although point estimates of RR started to diverge gradually as data became sparse, 90% and 81% of the estimated RRs covered the true RR in their 95% CIs when the data size was 1% and 0.25%, respectively (Figure 4C). MSEs remained small regardless of the number of cases per unit time and were comparable to those for the perfect control model (eFigure 8; http://links.lww.com/EDE/B425).

## DISCUSSION

In this study, we aimed to obtain robust estimates of the impact of PCV10 on pneumonia hospitalizations from sparse time series data. The synthetic control model was able to successfully adjust for underlying trends in the data when the control variables had relatively little noise. However, when the time series were sparse, the synthetic control model failed to adjust for unmeasured confounding and generated biased estimates of the impact of PCV10. These biases tended to become stronger as sample size decreased. This was particularly a problem when there was a strong secular trend, as was observed among the elderly in Brazil. As a possible solution, we decomposed the control variables and extracted the long-term trend, and then used this long-term trend as the control. This approach led to decreased bias in the estimates of the impact of PCV10.

Both the synthetic control and STL+PCA models found a decline in all-cause pneumonia hospitalizations among infants following introduction of PCV10, which was consistent with previous studies.^21–23^ Both of our models showed no changes in pneumonia hospitalizations after the introduction of PCV10 among the elderly in Brazil, while some previous studies found reductions among the elderly in the United States.^21,22^ Our previous reanalysis of the US data also found no decline in pneumonia in the elderly when using the synthetic control method but did find a decline when using simple linear trend adjustment.^7^ Therefore, the differences between studies are likely due to differences in how they control for unmeasured bias and confounding.

When the data did not have a long-term secular trend (i.e., among <12-month-old children in our study), the sample size did not affect the performance of the synthetic control model (eFigure 6A; http://links.lww.com/EDE/B425). It should be noted, however, that this is not because the model worked well in the young age group. In fact, similar to the old age group, the synthetic control model also failed to select an appropriate set of control diseases in this age group. However, due to the lack of a secular trend, the intercept-only model was able to generate a reasonable counterfactual in this instance. We suspected that the variable selection process in the synthetic control model did not work with the sparse data because the time series data became noisier, and many control diseases had zero cases or only a few cases per unit time in the most heavily down-sampled datasets. To test this hypothesis, we introduced the national-level control diseases in the synthetic control model in addition to the down-sampled control diseases. This process helped the synthetic control model to select an optimal set of control diseases, and as a result, the bias in estimated RRs was successfully corrected (eFigure 9; http://links.lww.com/EDE/B425). This analysis suggested that the problem of the variable selection may be attributed to the sparse data for control diseases but not the outcome. Not only the number of cases per unit time but also other characteristics of the time series change at local scales, such as the degree of autocorrelation and random epidemics happening in states. These different characteristics, whether due to measurement error, localized epidemics, or other issues related to scale, all contribute to the “noise” of the control diseases. This noise obscured the underlying long-term trend that was captured by both the outcome and control diseases and made it difficult to assess correlation between the outcome and control diseases. Effectively, this results in an “error-in-covariates” situation in the regression where the coefficients were biased toward zero,^24^ and thus, the synthetic control model failed to adjust for trends using the sparse control time series.

We proposed the STL+PCA model as a possible solution to the problem of data sparsity. The approach involves first extracting trends from the control time series, then using these smoothed trends to adjust pneumonia rates. Using both simulated and real-world data, we demonstrated that this alternative approach helps to reduce the impact of sparseness and to decrease bias in the estimates for smaller populations. The first step, STL decomposition, makes it easy to identify a long-term trend in noisy time series for control diseases. The second step, PCA, allows us to find a projection that explains the maximum variability of the outcome (i.e., PC1). Users can then simply fit a regression with PC1 and generate counterfactual for the postvaccine period. Both seasonal-trend decomposition and principal components analysis are widely used and readily available in the major statistical software. Similar to the synthetic control model, users can include all control diseases in this STL+PCA model, as long as the chosen controls satisfy the key assumptions (i.e., are not affected by the vaccine, and relationships with the outcome would not have changed had the vaccine not been introduced). An important disadvantage of the STL+PCA model is that it is no longer straightforward to interpret relationships between the outcome and control diseases, as the original time series for each control disease is not directly used as a covariate in the model. That is problematic if users want to understand associations between the outcome and control diseases but is less of a concern if one’s objective is to make a robust counterfactual and quantify the impact of vaccine. The choice of the set of control diseases has a different impact for the synthetic control model and STL+PCA model. Adding a noninformative control disease would have little impact on the synthetic control approach because it will not be selected; however, with the STL+PCA approach, if the long-term trend of the noninformative control has a large variance, it could change the rank of the principal components and move the relevant trends out of the first principal component.

One might argue that the Bayesian variable selection process should be allowed to choose a few appropriate control disease trends to be included in the regression model, instead of performing PCA and finding the “composite” of the trends. However, the model did not converge due to the strong collinearity among extracted trends, which made it difficult to use the Bayesian variable selection. One might also argue that we should omit the STL step and perform PCA with original time series for control diseases. This approach, however, did not generate a reliable counterfactual when the original data were noisier. We found that the STL step, which allowed us to isolate long-term trends from seasonality and the remainder component, was a key step to generate a robust counterfactual for sparse and noisy time series from small populations.

Another possible way to reduce the impact of sparseness is to aggregate monthly data into quarterly data and fit the synthetic control model. This simple process increases the number of observations per unit time, thereby allowing the synthetic control model to create an optimal composite of control diseases. This approach worked well with the Brazil data (eFigure 10; http://links.lww.com/EDE/B425). However, it may not be a good solution when the prevaccine data are limited. Using lower resolution time series reduces the number of data points, which makes it difficult to establish relationships between the outcome and the synthetic control. Alternative approaches could involve using a latent variable model to explicitly model the observation process, using a spatial model to borrow statistical information between adjoining localities, or using model stacking approaches.^25,26^

In conclusion, the STL+PCA method could be an effective tool to infer the causal impact of vaccines and other public health interventions and works well with sparse data. This model will enable us to quantify the impact of interventions more accurately, especially for small populations, and will allow us to address various public health questions using population health data that are readily available.

## ACKNOWLEDGMENT

The authors are grateful to the Department of Vital Statistics, a branch of the Brazilian Ministry of Health, for providing the hospitalization data.

## Supplementary Material
