# Supplementary material for: Challenges in Estimating the Impact of Vaccination with Sparse Data
Source: Epidemiology. 2018 Nov 30;30(1):61–8. doi: 10.1097/EDE.0000000000000938 (PMC6276862; doi:10.1097/EDE.0000000000000938)
Supplement: Supplementary file 1 [file ede-30-061-s001.pdf]

SUPPLEMENTAL DIGITAL CONTENT

eAppendix 1: Additional information on hospitalization data in Brazil

The national hospital discharge database covers the majority of the national population in Brazil (e.g., 82% in 2012).[1] Diseases were categorized using International Classification of Diseases (ICD) 10 code, and only the primary ICD10 discharge code was consistently available for each hospitalization event. Data from Brazil were selected in this study because it has one of the most comprehensively-collected hospitalization databases in the world and has high geographic resolutions. Brazil also has interesting geographic characteristics, capturing a broad range of income/development levels and climatic zones. There are 27 states and 5 regions in the country, which largely reflect climatic differences, as well as sub-national variations in human development, as based on income, longevity, and education. For the senior age group, two states in the North region were dropped from analysis, because ICD10 chapters included in the model had fewer than 10 hospitalizations per month on average in the study period. Therefore, 27 states were included in analysis for the young age group, while the old age group had 25 states. Pre-processing of the hospitalization data, such as an adjustment for the rapid shift in the number of hospitalizations due to the change in coding practice in 2008, was done as described in our previous study.[2]

eAppendix 2: Additional information on down-sampled data

To assess how the number of events in a time series may affect predictions from various quantitative models, we performed a down sampling analysis.[3,4] From the national-level data, we randomly subsampled the time series of ICD10 chapters, including all-cause pneumonia hospitalizations, using binomial distribution with various rates such that

$$Downsampled\_cases_{i,t} \sim \text{Binomial}(Observed\_cases_{i,t}, Rate)$$

where $Observed\_cases_{i,t}$ was the actual number of hospitalizations for disease *i* reported in time period *t* at the national-level. Cases were randomly sampled at rates of 10%, 1%, and 0.25%, to simulate the population sizes of different regions and states in Brazil. For example, the population sizes of the five regions were between 5% and 48% of the entire national population of infants and seniors. At the state level, the smallest state represented only 0.3% (State 14 in the North region; n=10,500) for children under 12 months of age and 0.2% (State 12 in the North region; n=5,900) of people 80+ years of age across Brazil.

We repeated this sampling process 100 times and created 100 down-sampled datasets at each of these rates. We then fit the models to each of these down-sampled datasets and quantified the impact of the vaccine. To evaluate the effect of sample size on the performance of the models, the estimated impact of the vaccine from the down-sampled data were compared to those from the national-level data, assuming the national estimate was the "ground truth."

eAppendix 3: Additional information on the simulated time series data

The number of outcome ($Outcome_t$) and four control diseases ($ControlA_t$, $ControlB_t$, $ControlC_t$, and $ControlD_t$) are randomly sampled from the following means to generate the time series:

$$\lambda_{outcome\_t} = \exp\,(\beta_0 - 1.5 * t_1 + 3 * spl_0 + \frac{\log(0.8)}{24} * spl_1 - \frac{\log(0.8)}{24} * spl_2 + 0.1 * sin\left(\frac{2\pi t}{12} + 0.2\right))$$

$$\lambda_{controlA\_t} = \exp\,(\beta_0 - 1.5 * t_1 + 3 * spl_0 + 0.1 * sin\left(\frac{2\pi t}{12} + 0.2\right))$$

$$\lambda_{controlB\_t} = \exp\left(0.965 * \beta_0 - 0.75 * t_1 + 1.5 * spl_0 + 0.1 * sin\left(\frac{2\pi t}{12} + 1.2\right)\right)$$

$$\lambda_{controlC\_t} = \exp\left(0.94 * \beta_0 - 1.8 * t_1 + 3.6 * spl_0 + 0.05 * sin\left(\frac{2\pi t}{12} + 0.4\right)\right)$$

$$\lambda_{controlD\_t} = \exp\left(\beta_0 - 0.3 * t_1 + 0.6 * spl_0 + 0.15 * sin\left(\frac{2\pi t}{12} + 0.8\right)\right)$$

spl_0 captures a secular trend that begins in month 43 (specified as a linear spline). spl_1 and spl_2 capture a vaccine-associated decline, which continues for 24 months and then levels out (also specified as linear splines).

$$Outcome_t \sim \text{Poisson}(\lambda_{outcome\_t})$$

$$ControlA_t \sim \text{Poisson}(\lambda_{controlA\_t})$$

$$ControlB_t \sim \text{Poisson}(\lambda_{controlB\_t})$$

$$ControlC_t \sim \text{Poisson}(\lambda_{controlC\_t})$$

$$ControlD_t \sim \text{Poisson}(\lambda_{controlD}\_t)$$

$$\beta_0 = \log(8000 * p)$$

$$t_1 = \frac{1}{120}, \frac{2}{120}, \dots, 1$$

where p is the sample size (1, 0.1, 0.01, or 0.0025). For each value of p, we simulated 100 time series data. $ControlA_t$ is considered "perfect control," as it had the exact same trend as the outcome until the simulated vaccine introduction.

eAppendix 4: Additional information on the synthetic control (SC) model

The SC modeling framework is as follows:

$$\ln(Y_t + 0.50) = \beta_0 + \sum_{j=2}^{12} \gamma_j I(m(t) = j) + \sum_{j=1}^{p} \beta_j(\delta_j) \ln(\mathrm{x}_{jt} + 0.50) + \epsilon_t;$$

$$t = 1,2,\ldots,\text{total number of time points}$$

where Y_t represents the number of pneumonia hospitalizations at time *t*; x_{jt} represents the count of control disease j at time *t*; $m(t)$ is a function that maps a time point to the corresponding calendar month; γ_j represents the month j regression coefficient; $I(.)$ represents the indicator function; p is the total number of control diseases in the analysis; $\beta_j(\delta_j)$ is the regression coefficient for control disease j which is given a spike-and-slab prior distribution (depending on δ_j) in order to allow for data-driven variable selection[5]; δ_j are binary random variables that indicate if control disease j is included in the regression model ($\delta_j = 1$) or not ($\delta_j = 0$); and $\epsilon_t \sim N(0, \sigma_\epsilon^2)$. The regression coefficients, $\beta_j(\delta_j)$ for control disease j, are not time varying, because we assume that the relationships between the outcome and control diseases are constant over time. Time series for the outcome and control diseases were log-transformed prior to being used in the model in order to alleviate the effects of epidemics on the long-term trend and to more closely resemble normality assumed in the model. As a continuity correction, 0.5 was added to all data points. The 2009 influenza pandemic was adjusted for by including dummy variables for the months in which the pandemic peaked.[1] The full list of control diseases included in the SC model can be found in eTable 1. Control diseases included in the model varied across states, because data on some control diseases might not be available or were filtered out because there were fewer than 10 hospitalizations per month on average in the study period in some states (See eAppendix 1 about filtering). On average, 15 and 18 control diseases were included for variable selection in the SC model in the states among children and the elderly, respectively.

Full details on the prior distribution can be found in the paper by Bruhn, *et al.*[1,5] Briefly, we used spike and slab priors to select variables in the candidate models with equal prior probability of inclusion for each variable (π = 0.5). We collected 9,000 posterior samples after a

burn-in period of 1,000 iterations for each fitted model. The convergence of the Markov chain Monte Carlo sampling algorithm was evaluated using the Geweke diagnostic and Gelman Rubin diagnostic.[6-8] There were no obvious signs of non-convergence as the p-values from the Geweke diagnostic tests were all larger than 0.05 and the potential scale reduction factors were <1.1 across all parameters. The effective sample size was also checked to confirm that we had enough posterior samples to make valid inference.[9,10]

eAppendix 5: Additional information on the STL+PCA model

The seasonal-trend decomposition procedure based on locally weighted scatterplot smoothing (STL method) decomposes time series into three components: trend, seasonality, and the remaining variation in data (eFigure 3).[11] The observed number of control disease j cases in month *t*, denoted by $ControlDisease_{jt}$, can be written as follows:

$$\ln(ControlDisease_{jt} + 0.50) = T_{jt} + S_{jt} + R_{jt}$$

where T_{jt}, S_{jt}, and R_{jt} are the trend component, annual seasonal component, and remainder component, respectively. For the STL trend extraction, the span of the locally weighted scatterplot smoothing (LOESS) window can be changed to control the smoothness of the extracted trends. The larger the LOESS window is, the smoother the extracted trends are (eFigure 4). We set it to be 5, 25, and 59 months, and selected the optimal size using the deviance information criterion (DIC).[12] The model with the optimal window size was then used to generate the counterfactual for the post-vaccine period and to quantify the impact of vaccine.

The principal component analysis (PCA) allows us to create new uncorrelated projections that explain the maximum variability in the data overall.[13-16] PCA was applied to the trends for control diseases extracted by the STL method. Extracted trends were first converted into standard

deviation units (Z scores) by subtracting off the mean of each variable and dividing by its standard deviation, as the variables included in PCA need to have the same scale. PCA involved an $n \times p$ matrix, X, where p represents the number of control diseases and n represented the total number of time points. The jth column of matrix X, x_j, represents the time series of a scaled extracted trend for control disease j. We then find a linear combination of these extracted trends (i.e., columns of matrix X) with maximum variance, which are given by

$$\sum_{j=1}^{p} a_j x_j = Xa$$

where a is a vector of constants $a_1, a_2, \dots, a_p$. The variance of this linear combination can be written as follows:

$$Var(Xa) = a'Sa$$

where S represents the sample covariance matrix associated with the dataset. Therefore, the linear combination that explains the largest variance can be identified by obtaining a p-dimensional vector a which maximizes the quadratic form $a'Sa$.

The JAGS model was fit using the rjags package in R version 3.4.3 (Vienna, Austria).[17] We initialized two independent Markov chains and collected 10,000 posterior samples after a burn-in period of 5,000 iterations for the Brazil data, and 5,000 posterior samples after a burn-in period of 1,000 iterations for the simulated time series data. Convergence was assessed as described in eAppendix 4. The JAGS model was specified as follows. A negative binomial regression was used due to the over-dispersion present in the data:

$$\lambda_t = \exp\left(\beta_0 + \sum_{j=2}^{12} \gamma_j I(m(t) = j) + \beta_1 PC1_t\right)$$

$$PrevaccineOutcome_t \sim \text{Negative Binomial}(p_t, \sigma)$$

$$p_t = \frac{\sigma}{\sigma + \lambda_t}$$

$$\sigma \sim \text{Uniform}(0, 50); t = 1,2, \ldots, total\ number\ of\ time\ points$$

$$\gamma_j \sim N(0, 0.00001); j = 2, \ldots, 12$$

$$\beta_k \sim N(0, 0.00001); k = 0, 1$$

where $PrevaccineOutcome_t$ represents the number of pneumonia hospitalizations at time *t* in the pre-vaccine period; $\mathrm{p_t}$ is a probability parameter specific to time period *t*; $m(t)$ is a function that maps a time point to the corresponding calendar month; γ_j represents the month *j* regression coefficient; $I(.)$ represents the indicator function; β_0 is an intercept; β_1 is the regression coefficient for the first principal component (PC1); σ is a dispersion parameter for the negative binomial model; λ_t is the expected value of the negative binomial distribution; and $\epsilon_t \sim N(0, \sigma_\epsilon^2)$.

eAppendix 6: Results on cross validation of the STL+PCA model

We have performed cross validation using the down-sampled pre-vaccine data with the rate 0.25% for the elderly in Brazil. We trained our model using four years of pre-vaccine data (2004–2007) and generated prediction for 2008. Although it was part of the pre-vaccine period, we avoided using the 2009 data due to a large 2009 influenza outbreak. The 95% credible interval of the rate ratio included one in 97% of 100 down-sampled datasets, suggesting that this model successfully predicted the 2008 data.

eAppendix 7: Full list of posterior inclusion probabilities of control diseases for children <12 months of age

Please see the Excel file.

eAppendix 8: Full list of posterior inclusion probabilities of control diseases for adults 80+ years of age

Please see the Excel file.

eAppendix 9: Mean squared error, variance, and bias squared

The performance of models was compared using the mean squared error (MSE), which was calculated as follows:

$$MSE = \frac{\sum_{i=1}^{n}\left(National\ RR - RR_i\right)^2}{n}$$

where RR_i represents a rate ratio for dataset *i* and *n* is the number of total datasets compared in each analysis (i.e., total number of states for state-level analysis and 100 for down-sampled analysis).

MSE was decomposed to variance and bias squared, which were calculated as follows:

$$Variance = \frac{\sum_{i=1}^{n}(RR_i - \overline{RR})^2}{n}$$

$$Bias^2 = (\overline{RR} - National\ RR)^2$$

where $\overline{RR} = \frac{\sum_{i=1}^{n}(RR_i)}{n}$. The sum of variance and bias squared is equal to MSE.

eTable 1. List of Control Diseases Included in the Synthetic Control Model.

Grouping scheme	ICD-10		Exclusions
**ICD-10 chapters**			
	C00_D48	Neoplasms	
	D50_89	Diseases of blood and blood-forming organs and certain disorders involving the immune mechanism	
	E00_99	Endocrine, nutritional, metabolic disorders	
	G00_99_SY	Diseases of the nervous system	G00_04
	H00_99_SY	Diseases of the ear and mastoid process	H10, H65, H66
	I00_99	Diseases of the circulatory system	
	K00_99	Diseases of the digestive system	
	L00_99	Diseases of the skin	
	M00_99	Diseases of the musculoskeletal system	
	N00_99	Diseases of the genitourinary system	
	P00_99	Perinatal diseases	
	Q00_99	Congenital malformations, deformations and chromosomal abnormalities	
	S00_T99	Injury, poisoning and consequences of external causes	
	Z00_99	Factors influencing health status and contact with health workers	
**Other grouped outcomes**			
	a10_b99_nopneumo	Certain infectious and parasitic diseases, except intestinal	A40.3, B95
	B20_24	HIV	
	E10_14	Diabetes	
	E40_46	Malnutrition	
	I60_64	Stroke	
	J20_22	Bronchitis, bronchiolitis and unspecified acute lower respiratory infection	
	P05_07	Premature delivery and low birth weight	
	ach_noj	All nonrespiratory hospitalizations	J00_99, F and O chapters
**Specific outcomes**			
	A39	Meningococcal infection	
	A41	Other septicemia	
	B34	Viral infection of unspecified site	
	K35	Appendicitis	
	K80	Cholelithiasis	
	N39	Urinary tract infection	

eFigure 1. National-level Time Series of Observed and Counterfactual All-cause Pneumonia Hospitalizations Generated by the Synthetic Control Model for (A) Cases <12 Months and (B) 80+ Years of Age in Brazil.

A) <12 months

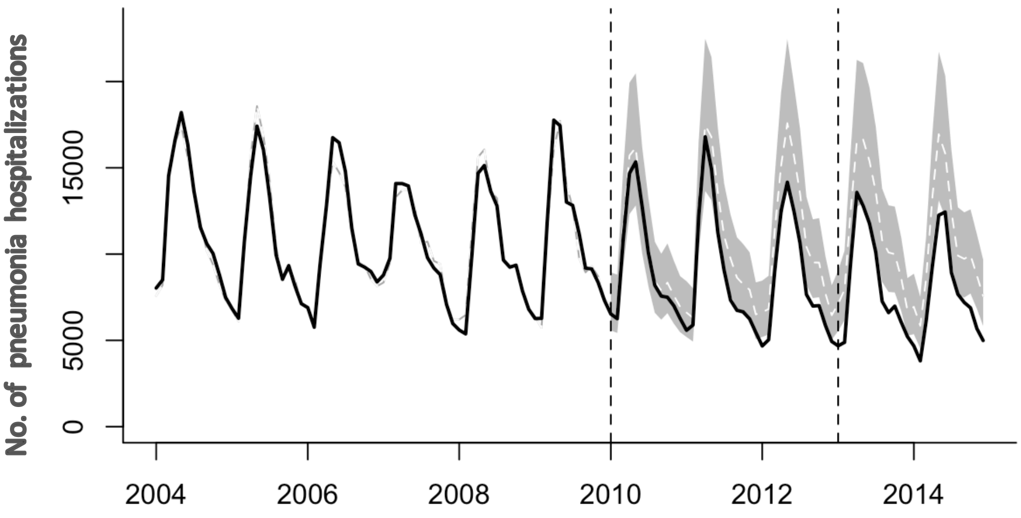

B) 80+ years

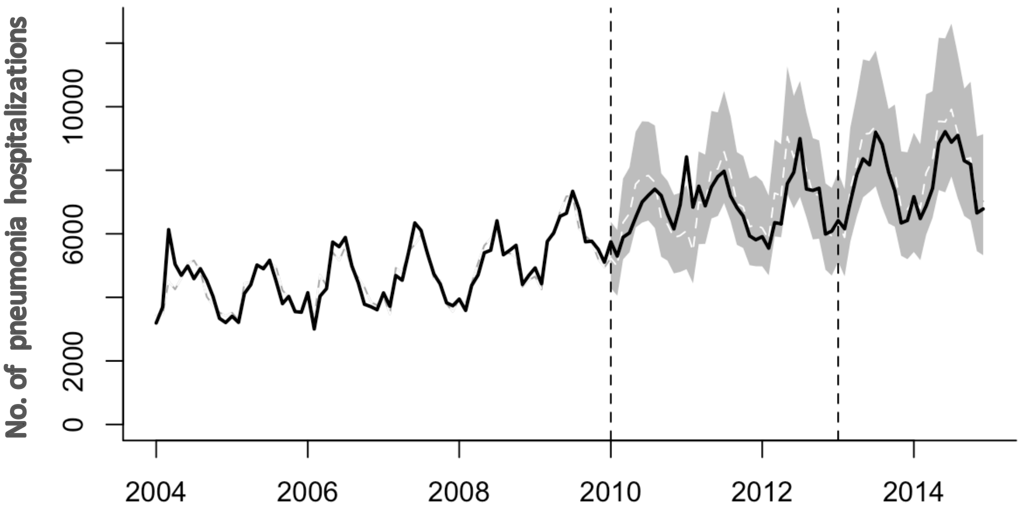

The observed number of pneumonia hospitalizations is represented by black lines. The counterfactual number of pneumonia hospitalizations and their 95% credible intervals are represented by white dashed lines and grey areas. Vertical dashed lines indicates the timing of PCV10 introduction (January 2010) and the start of the evaluation period (January 2013).

Abbreviations: PCV10, 10-valent pneumococcal conjugate vaccine.

eFigure 2. Simulated Monthly Time Series Data From a Single Simulation.

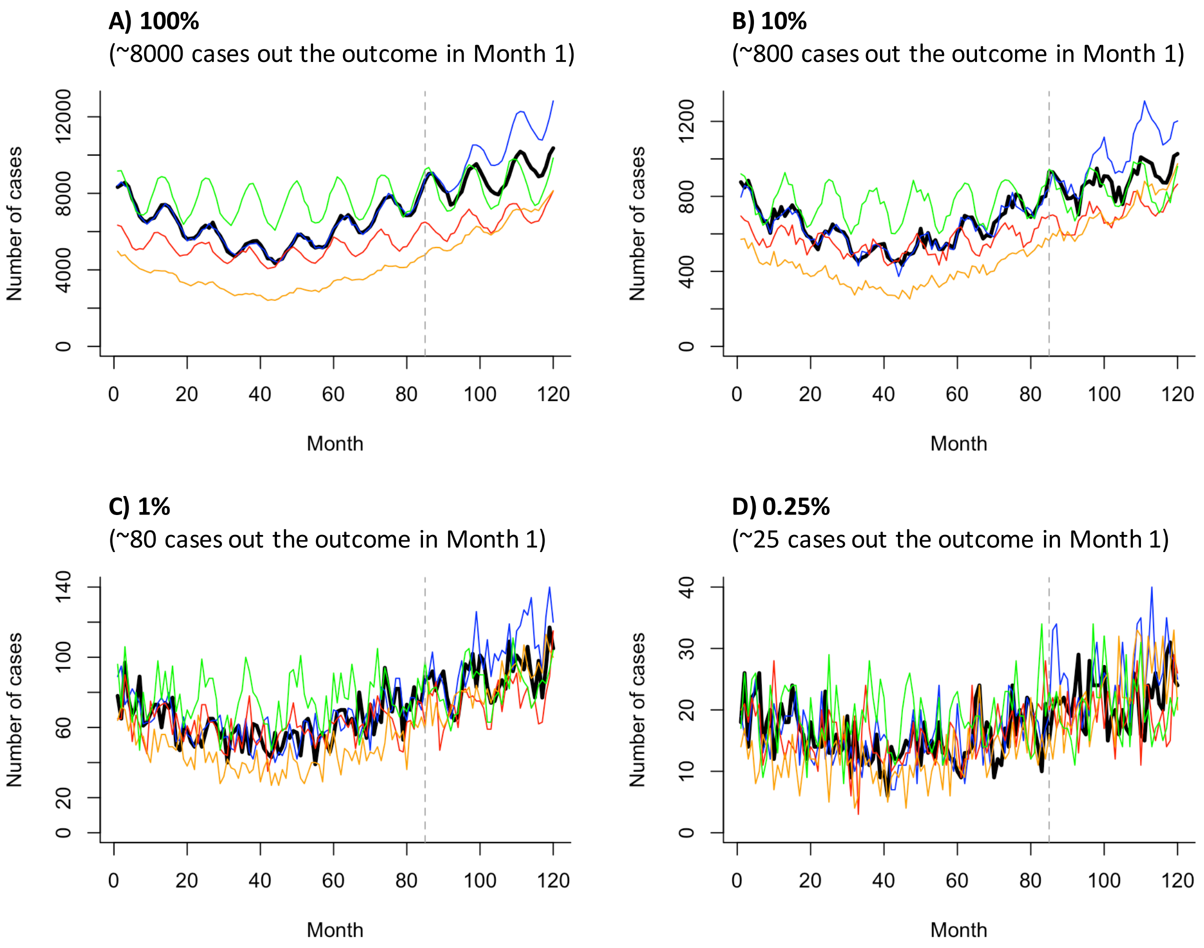

The outcome is in black, the perfect control is in blue, and the remaining controls are in green, orange, and red. Percentages at the top of panels represent the sample size. Vertical dashed lines represent the timing of the simulated vaccine introduction (Month 85).

eFigure 3. Diagram of the STL+PCA Model.

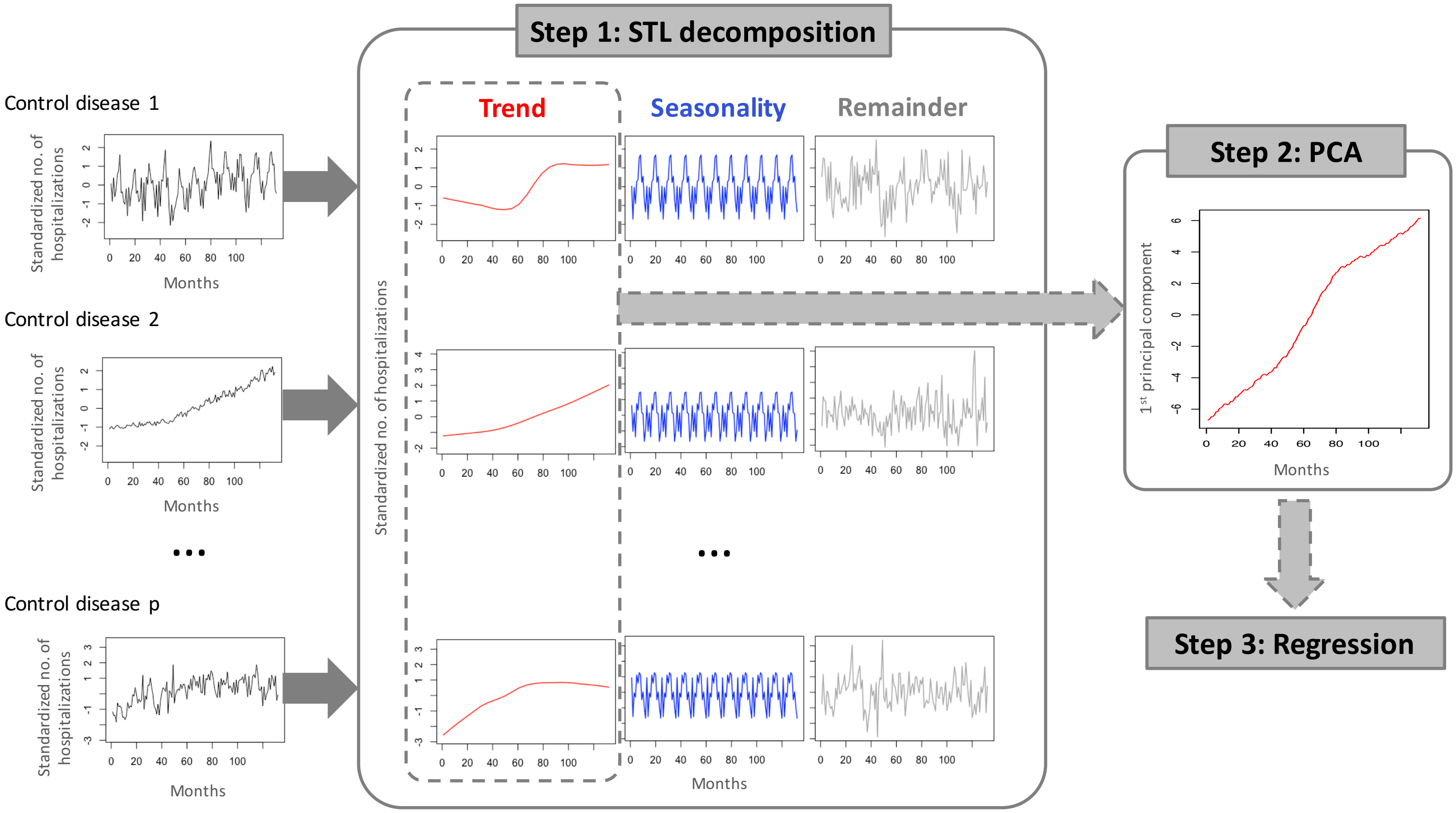

Abbreviations: STL, seasonal-trend decomposition procedure based on locally weighted scatterplot smoothing; PCA, principal component analysis.

eFigure 4. Example of STL Decomposition Using Different Sizes of the LOESS Window (ICD10 code: I00_99, 80+ yo in Brazil).

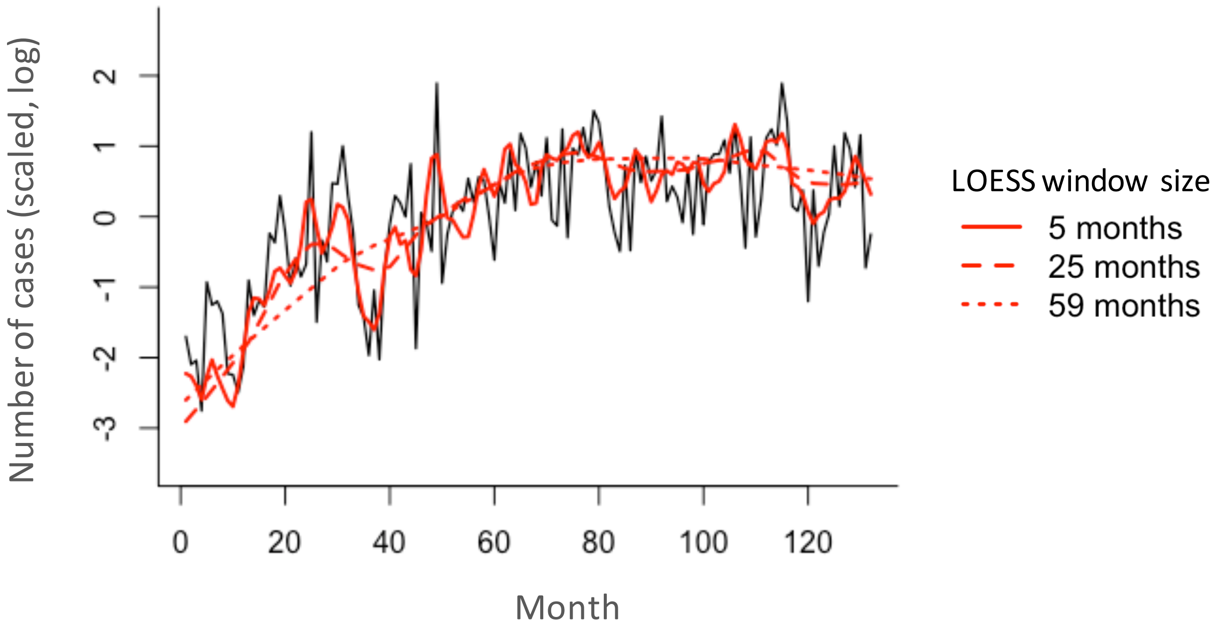

Observed number of hospitalizations is in black, and trends extracted by the STL method using different sizes of the LOESS window (5, 25, and 59 months) are in red.

Abbreviations: LOESS, locally weighted scatterplot smoothing; STL, seasonal-trend decomposition procedure based on locally weighted scatterplot smoothing.

eFigure 5. Variance (A) and Bias Squared (B) of Estimated Rate Ratios from Down-sampled Datasets (80+ yo, Brazil).

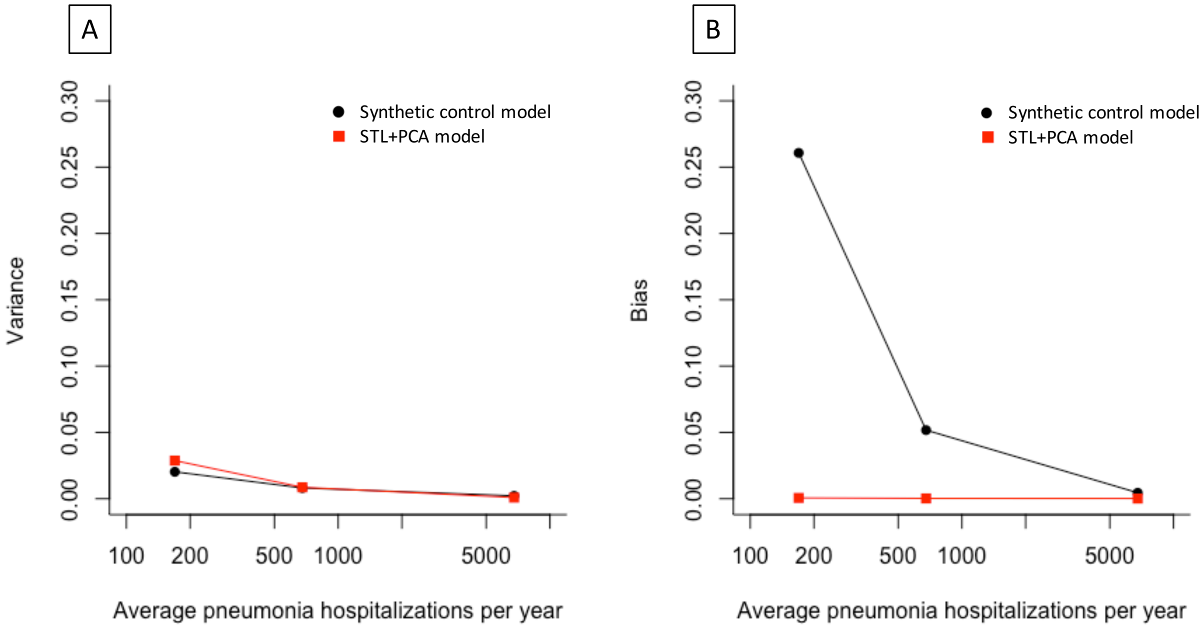

Abbreviations: STL, seasonal-trend decomposition procedure based on locally weighted scatterplot smoothing; PCA, principal component analysis.

eFigure 6. Estimated Rate Ratios for Down-sampled Datasets (<12 mo, Brazil).

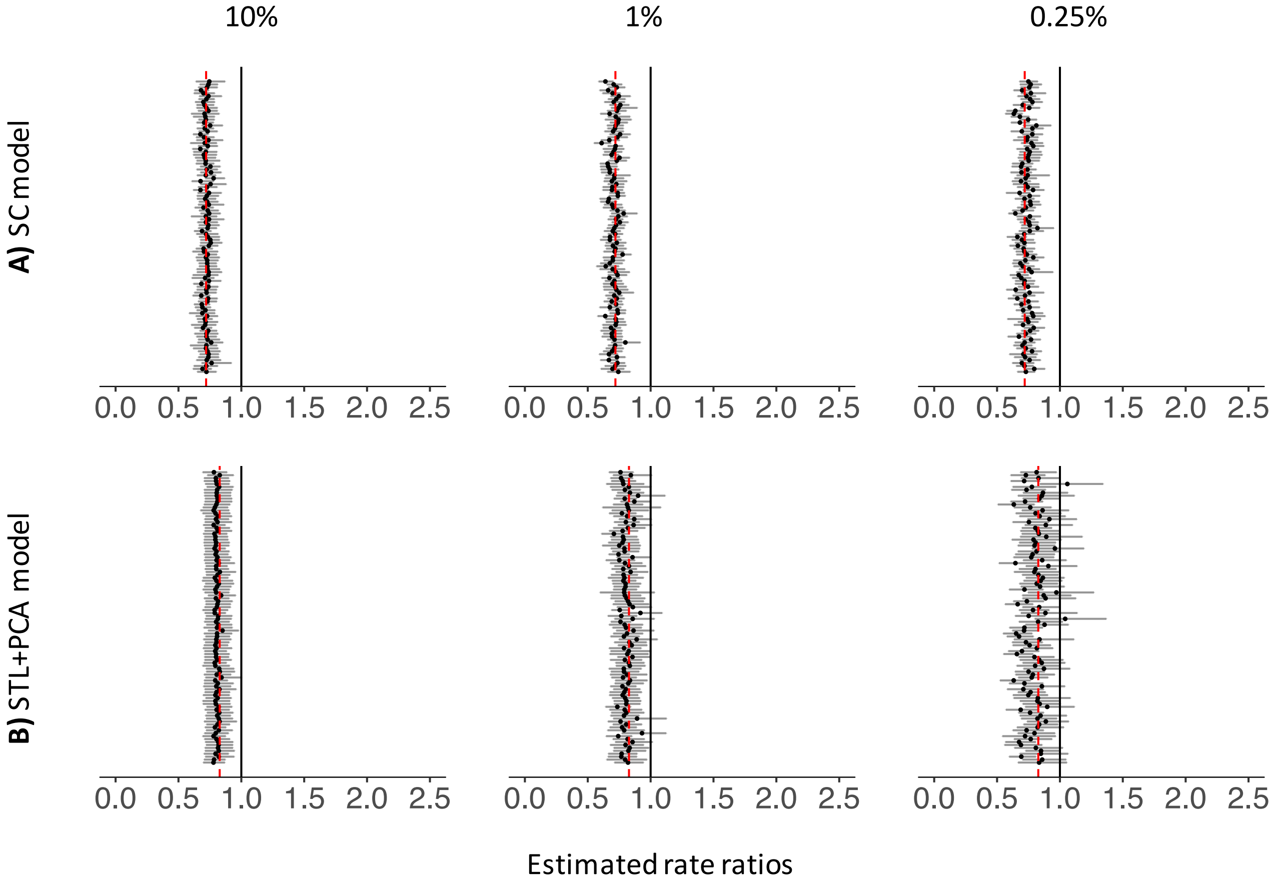

Each black dot represents a RR estimated for each down-sampled dataset. Dark grey bars associated with these dots represent 95% credible intervals for RRs. The percentages at the top represent the down-sampling rates. Black vertical lines represent the null value (RR=1) and red dashed lines represent national estimates of RR generated by each type of the model.

Abbreviations: RR, rate ratio; SC, synthetic control; STL, seasonal-trend decomposition procedure based on locally weighted scatterplot smoothing; PCA, principal component analysis.

eFigure 7. Mean Squared Errors of Estimated Rate Ratios from Down-sampled Datasets (<12 mo, Brazil).

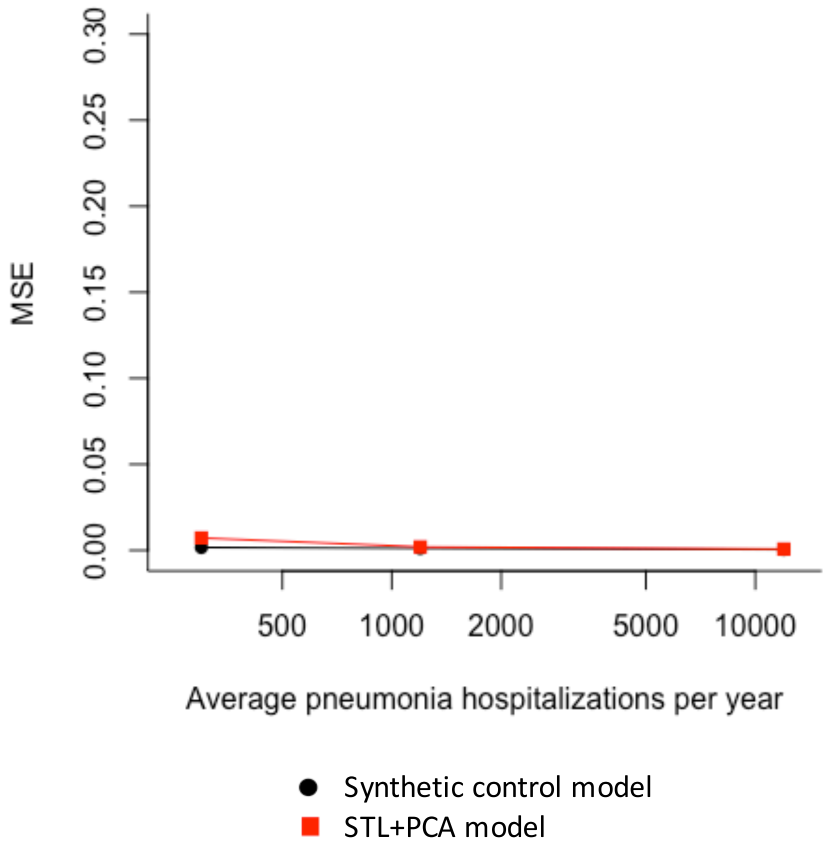

Abbreviations: MSE, mean squared error; STL, seasonal-trend decomposition procedure based on locally weighted scatterplot smoothing; PCA, principal component analysis.

eFigure 8. Mean Squared Errors (A), Variance (B), and Bias Squared (C) of Estimated Rate Ratios from Simulated Time Series Data.

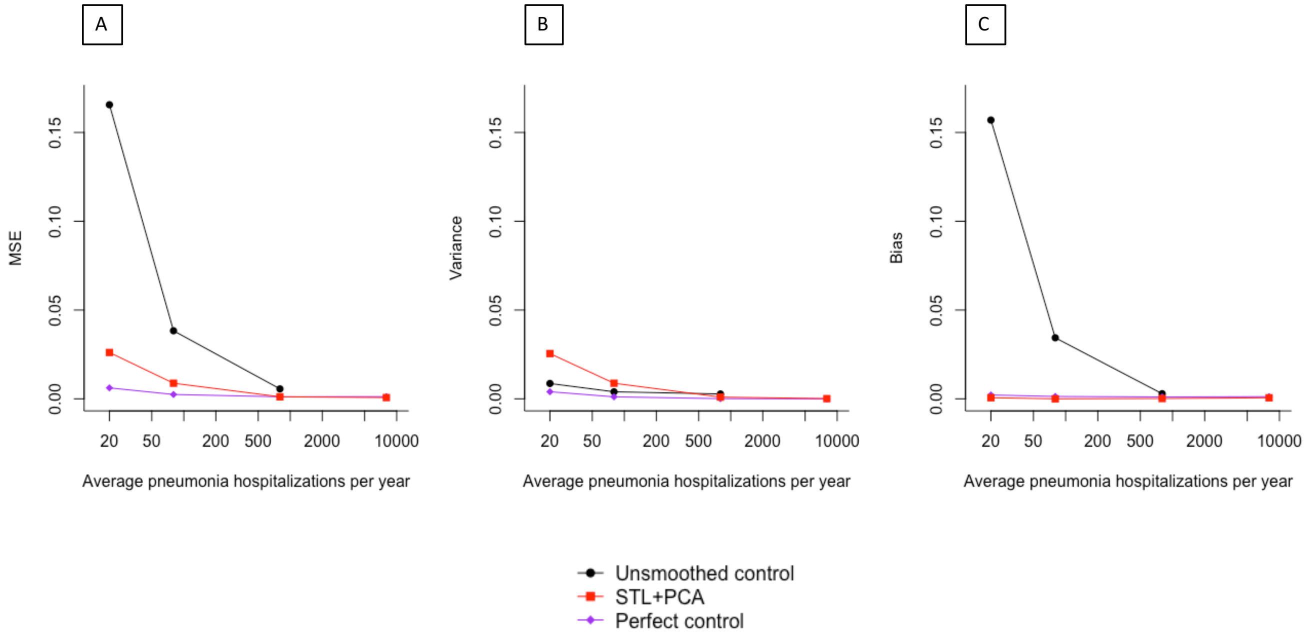

Abbreviations: MSE, mean squared error; STL, seasonal-trend decomposition procedure based on locally weighted scatterplot smoothing; PCA, principal component analysis.

eFigure 9. Rate Ratios for Down-sampled Datasets Estimated by the Synthetic Control Model Including Both National-level Covariates and Down-sampled Covariates (80+ yo, Brazil).

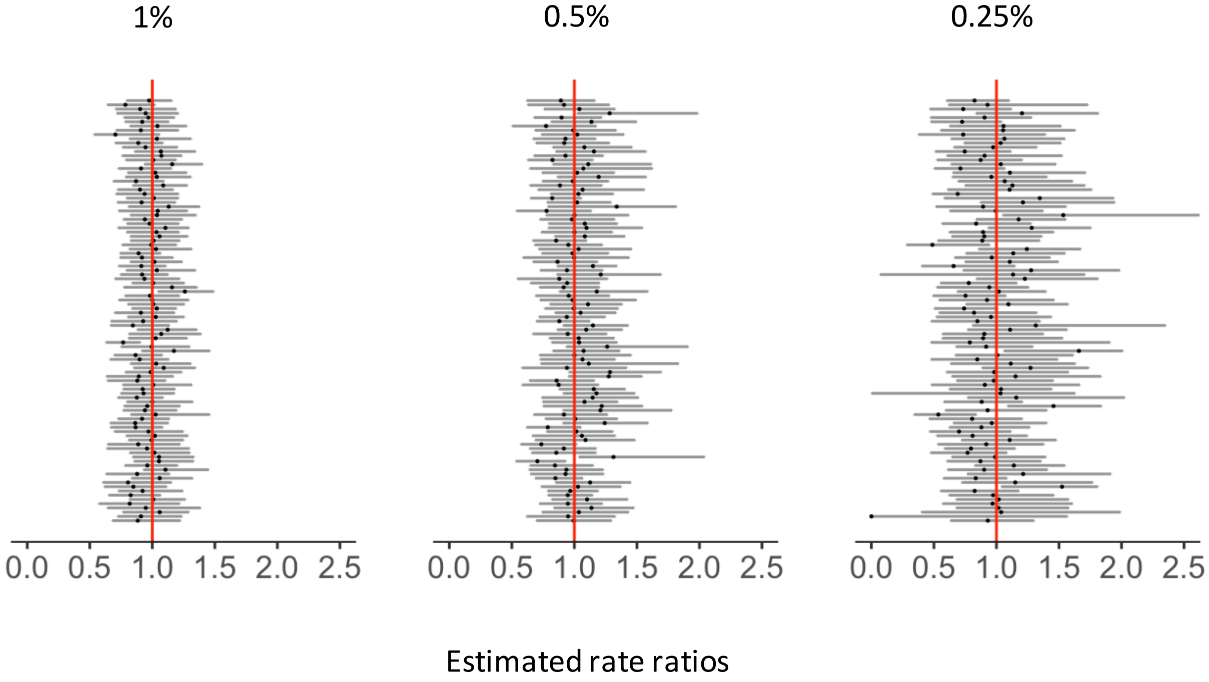

Each black dot represents a rate ratio (RR) estimated for each down-sampled dataset. Dark grey bars associated with these dots represent 95% credible intervals for RRs. Vertical red lines represent the null value (RR=1). The percentages at the top represent the down-sampling rates.

eFigure 10. Rate Ratios for Down-sampled Datasets Estimated by the Synthetic Control Model Using Quarterly Data for 80+ yo, Brazil.

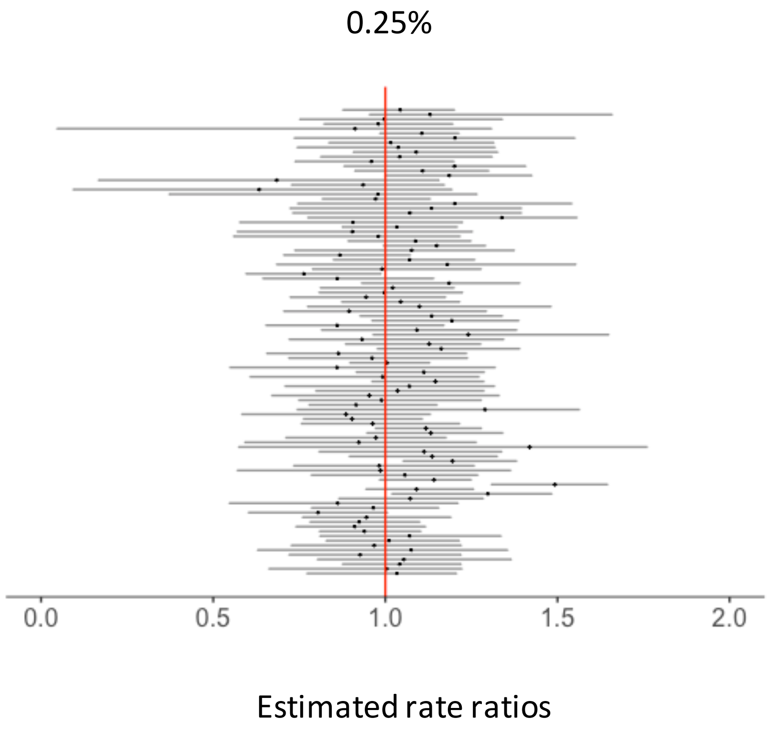

Each black dot represents a rate ratio (RR) estimated for each down-sampled dataset. Dark grey bars associated with these dots represent 95% credible intervals for RRs. Vertical red lines represent the null value (RR= 1). The percentages at the top represent the down-sampling rates.

REFERENCES

1. Bruhn CA, Hetterich S, Schuck-Paim C, Kurum E, Taylor RJ, Lustig R, Shapiro ED, Warren JL, Simonsen L, Weinberger DM. Estimating the population-level impact of vaccines using synthetic controls. *Proc Natl Acad Sci U S A* 2017;**114**(7):1524-1529.
2. Schuck-Paim C, Taylor RJ, Simonsen L, Lustig R, Kurum E, Bruhn CA, Weinberger DM. Challenges to estimating vaccine impact using hospitalization data. *Vaccine* 2017;**35**(1):118-124.
3. Viboud C, Bjornstad ON, Smith DL, Simonsen L, Miller MA, Grenfell BT. Synchrony, waves, and spatial hierarchies in the spread of influenza. *Science* 2006;**312**(5772):447-51.
4. Nelson MI, Lemey P, Tan Y, Vincent A, Lam TT, Detmer S, Viboud C, Suchard MA, Rambaut A, Holmes EC, Gramer M. Spatial dynamics of human-origin H1 influenza A virus in North American swine. *PLoS Pathog* 2011;**7**(6):e1002077.
5. Brodersen KH, Gallusser F, Koehler J, Remy N, Scott SL. Inferring causal impact using Bayesian structural time-series models. *The Annals of Applied Statistics* 2015;**9**(1):247-274.
6. Geweke J. *Evaluating the accuracy of sampling-based approaches to the calculation of posterior moments*. Vol. 196 Federal Reserve Bank of Minneapolis, Research Department Minneapolis, MN, USA, 1991.
7. Gelman A, Rubin DB. Inference from iterative simulation using multiple sequences. *Statistical science* 1992:457-472.
8. Brooks SP, Gelman A. General methods for monitoring convergence of iterative simulations. *Journal of computational and graphical statistics* 1998;**7**(4):434-455.
9. Ripley BD. *Stochastic simulation*. Vol. 316 John Wiley & Sons, 2009.

10. Kass RE, Carlin BP, Gelman A, Neal RM. Markov chain Monte Carlo in practice: a roundtable discussion. *The American Statistician* 1998;**52**(2):93-100.

11. Cleveland RB, Cleveland WS, Terpenning I. STL: A seasonal-trend decomposition procedure based on loess. *Journal of Official Statistics* 1990;**6**(1):3.

12. Spiegelhalter DJ, Best NG, Carlin BP, Van Der Linde A. Bayesian measures of model complexity and fit. *Journal of the Royal Statistical Society: Series B (Statistical Methodology)* 2002;**64**(4):583-639.

13. Pearson K. LIII. On lines and planes of closest fit to systems of points in space. *The London, Edinburgh, and Dublin Philosophical Magazine and Journal of Science* 1901;**2**(11):559-572.

14. Hotelling H. Analysis of a complex of statistical variables into principal components. *Journal of educational psychology* 1933;**24**(6):417.

15. Abdi H, Williams LJ. Principal component analysis. *Wiley interdisciplinary reviews: computational statistics* 2010;**2**(4):433-459.

16. Jolliffe IT, Cadima J. Principal component analysis: a review and recent developments. *Phil. Trans. R. Soc. A* 2016;**374**(2065):20150202.

17. Martyn Plummer. rjags: Bayesian Graphical Models using MCMC. R package version 4-6. 2016.
